# Making Sense of Adopted Children's Internal Reality Using Narrative Story Stem Techniques: A Mixed-Methods Synthesis

**DOI:** 10.3389/fpsyg.2018.01189

**Published:** 2018-07-10

**Authors:** Eileen Tang, Dries Bleys, Nicole Vliegen

**Affiliations:** Department of Clinical Psychology, KU Leuven, Leuven, Belgium

**Keywords:** adopted children, narrative story stem techniques, attachment representations, mixed-methods synthesis, tailored treatment

## Abstract

**Background:** Extant research on adopted children has consistently shown that early adverse experiences confer vulnerability to myriad developmental problems, which may be mitigated by the “natural intervention” of adoption itself and/or by treatment efforts. Narrative Story Stems Techniques (NSSTs) have been used in research and clinical practice to assess adopted children's developmental profiles in middle childhood. However, no study to date has systematically reviewed this body of literature.

**Objectives:** This paper presents a systematic review of research using NSSTs to make sense of adopted children's internal reality (i.e., perceptions, experiences, and representations), in terms of exploring theoretical perspectives as well as critically synthesizing findings and discussing implications.

**Methods:** State-of-the-art PRISMA guidelines were followed throughout, resulting in the identification of 18 records, comprising six qualitative, 10 quantitative, and two mixed-methods primary papers, reporting on seven unique studies. All records were assessed with regard to methodological quality. Data were extracted and synthesized narratively using an integrated design for mixed-methods synthesis.

**Results:** The findings suggest that, although NSST research with adopted children is still in its infancy, there is relatively robust evidence supporting the use of these techniques to assess and track developmental change in adopted children's attachment representations. In this regard, the non-verbal (aside from the verbal) approach to children's complex internal reality, as well as a more fine-grained (aside from a categorical or dimensional) perspective on children's NSST responses, are highlighted as particularly valuable in tailoring treatment to a particular child's needs and vulnerabilities. Moreover, several promising avenues for future research and clinical application of NSSTs, including the extension to affect-regulatory and mentalizing perspectives, may further our knowledge and understanding of, and thus treatment efforts toward, these often vulnerable children. However, these findings should be interpreted with caution, due to the limited number of studies characterized by considerable methodological heterogeneity.

**Conclusions:** In light of the findings of the present review, we strongly advocate future studies using NSSTs in theoretically and empirically consistent ways, in order to gain a better understanding of adopted children's internal reality in terms of attachment representations, affect-regulatory strategies, and mentalizing processes, and to track changes therein.

## Introduction

Adopted children have suffered at least one discontinuity in their caregiving environment. For whatever reason, they were separated from their primary caregivers to be adopted. For some of these children, this reason comprised the adverse events which they experienced at the hands of their primary caregivers. Moreover, for children adopted in an international context, most spent some time in residential care (Juffer and Tieman, 2009), some of which is of questionable quality. Such early adverse experiences have been shown to confer vulnerability to myriad developmental problems, ranging from insecure or disorganized attachment representations (e.g., Chisholm et al., 1995; van den Dries et al., 2009), deficits in emotion regulation, and difficulties maintaining peer relationships, to severe behavioral or educational problems (Perry, 2002, 2004; Anda et al., 2006). Once removed from the adverse environment, many of these children catch up to some degree (van Ijzendoorn and Juffer, 2006), due to the “self-righting” capacities of humans (Cicchetti and Rogosch, 1997) and the “good enough” environment of their new homes (Steele et al., 2010; Barone and Lionetti, 2012; Pace et al., 2012). However, alongside developmental progress and catch-up, developmental problems often persist, requiring help and treatment for the child and family.

To enable clinicians to tailor treatment to each adopted child's specific developmental profile, assessment methods that allow detailed exploration of a child's internal reality (i.e., perceptions, experiences, and representations) are needed. To this end, narrative story stem techniques (NSSTs) have been used in research and clinical practice. This generic term was first coined by Page (2001) to refer to narrative methods in which children are asked to complete story stems introduced by an interviewer. These methods have been developed to allow detailed examination of the child's representations of family life, which have been hypothesized to play a role in the mediation of the effects of early adversity on later development, and thus offer ports of entry for change and development (Hodges and Steele, 2000; Hodges et al., 2005).

NSSTs typically consist of a series of “stems,” the beginnings of stories describing everyday family scenarios, each of which contains an inherent dilemma (e.g., emotional upset, physical hurt, separation from parents, parental discipline and rejection/exclusion). For instance, in the “spilled juice” stem the family is seated around the table to drink juice, when the child protagonist accidentally knocks over the jar as s/he reaches for more juice. The stems are simultaneously spoken and played out with a standard set of family figures, animals, and play props. The child is then invited to “show me and tell me what happens next” (e.g., Hodges and Steele, 2000; Page et al., 2008). Interviews are videotaped and recordings are transcribed, to produce a script consisting of what the child says (the verbal narrative), what the interviewer says, and what the child does (the non-verbal narrative). Subsequently, children's narrative responses are coded for themes of interest. Currently, several approaches to coding NSSTs are used, most of which code elements of narrative content as well as overall structural characteristics of stories (for a review of scoring systems, see Page, 2001). Coding of children's story completions can thus reveal aspects of representations or scripts regarding social relationships, as well as affective and behavioral regulation processes (Robinson, 2007). Hence, NSSTs may prove useful in gaining important insights into adopted children's inner experiences from a theoretical, empirical, and clinical point of view. To date, extant research using NSSTs with adopted children has not been systematically reviewed. The present review aims to fill this gap.

Here, we set the stage by elaborating on why an attachment perspective is central to understanding adoption and developmental processes in adopted children. After describing the methodology used to conduct this systematic review, we provide a systematic, critical, and integrated overview of the quantitative and qualitative findings of primary studies that have attempted to make sense of adopted children's internal reality using NSSTs. We conclude with a discussion of the current evidence base for the use of NSSTs with adopted children, and highlight promising avenues for research and clinical application.

### Adoption in an attachment perspective

Attachment experiences (i.e., day-to-day interactive experiences between child and caregiver) become organized in memory in *internal working models* (IWMs; Bowlby, 1973, 1982) from infancy onwards. Through accumulated experience with specific caregivers the developing child internalizes experiences of being responded to by the caregiver and learns about the predictability of available care and protection. IWMs tend to become automatic, increasingly operating outside conscious awareness (Hodges et al., 2003), allowing the child to interpret and predict the caregiver's behavior, and to plan immediate and future responses. IWMs, as basic scripts for human relationships, thus exert a powerful influence on the child's understanding of current experiences, as well as his/her expectations of and reactions to new interactions and experiences (Hodges and Steele, 2000; Hodges et al., 2003, 2005). When children's needs for care have been adequately met, they develop *secure* attachment representations or IWMs (Page et al., 2008). Conversely, when children experience significantly inadequate care, as is the case for adopted children with a history of early adverse experiences, they are likely to develop *insecure* or *disorganized* IWMs (Carlson et al., 1989; Chisholm et al., 1995; Jaffari-Bimmel et al., 2006; Page et al., 2008). In this context, the meta-analysis of van den Dries et al. (2009) found that adopted children with a history of institutionalization showed less attachment security and more disorganized attachments, as assessed with observational but not self-report measures, than their non-adopted peers.

Although extreme insecure attachments in childhood have been associated with a wide range of developmental problems later in life (Howe, 2001; Hushion et al., 2006; Rutter et al., 2007; Page et al., 2008), research also suggests there is potential to catch up at cognitive and emotional levels (Dozier, 2005; Juffer and van Ijzendoorn, 2007; Bick and Dozier, 2008). These seemingly contradictory findings originate in the inherent nature of IWMs, which are characterized by both relative endurance and relative mutability (Page et al., 2008). IWMs can be revised over time in relation to significant changes in the caregiving environment (Steele et al., 2008, 2010; Pace et al., 2012). In this regard, several authors have described adoption as a “natural intervention” (e.g., van Ijzendoorn and Juffer, 2006). Adoption and the accompanying new attachment relationships are the most dramatic of any interventions that can help the child revise their early insecure or disorganized IWMs and alter the developmental course of children who have suffered traumatic experiences in early life (Schofield and Beek, 2005; Jaffari-Bimmel et al., 2006; Steele et al., 2007a; Pace et al., 2012). The new attachment relationships within the adoptive family develop in the interplay between what the child brings in terms of internalized early relationship experiences (i.e., IWMs) and what the adoptive caregivers bring in terms of their own expectations and attachment histories (Steele et al., 2003).

Researchers and clinicians are seeking ways to accurately assess change in aspects of adopted children's internal reality, including representations of attachment relationships. Although observational-behavioral measures (e.g., the Strange Situation; Ainsworth et al., 1978) or parent-report questionnaires (e.g., Attachment Security Questionnaire; Chisholm et al., 1995) also provide information about children's IWMs, these measures are limited in two crucial ways. First, there is a surprising lack of measures developed to specifically assess IWMs in middle childhood, although from a developmental perspective IWMs can undergo meaningful changes during this period (e.g., Bosmans and Kerns, 2015). Second, although these measures provide more general information about IWMs in terms of attachment classifications and/or dimensions, the question arises whether such general classifications/dimensions can provide practitioners with sufficiently detailed information about the adopted child's development to guide treatment. Several interview measures (e.g., Friends and Family Interview; Steele and Steele, 2005) are used in research and clinical practice to assess children's attachment representations. However, these measures are mostly applicable from age 8 onwards, plausibly because interviews rely heavily on children's explicit mentalizing (Fonagy and Luyten, 2009) and verbal-expressive skills, capacities that may be slower to develop in (internationally) adopted children with a history of early adversity. Furthermore, in interviews children are asked directly to talk about real (past or current) relationships, leaving them little room for escape when this theme proves too arousal- or anxiety-provoking or intrusive.

## Methods

### Aim and design

This review aims to identify studies making use of NSSTs to understand adopted children's internal reality and to synthesize the results of these studies. The objectives include exploring theoretical perspectives, summarizing empirical findings, and discussing clinical implications. To this end, a mixed-methods systematic literature review was conducted, following the PRISMA guidelines (Moher et al., 2009) throughout.

### Eligibility criteria

Studies were considered eligible if they conformed to following SPIDER criteria, as advocated by Cooke et al. (2012):

Sample: adopted children aged 3^1^–11;
Phenomenon of Interest: internal reality;
Design: NSST;
Evaluation: aspects of children's internal reality;
Research type: qualitative, quantitative, and mixed.

Additionally, only studies published in English-language peer-reviewed journals or books were considered for inclusion in this review.

### Search strategy

**Figure 1:**
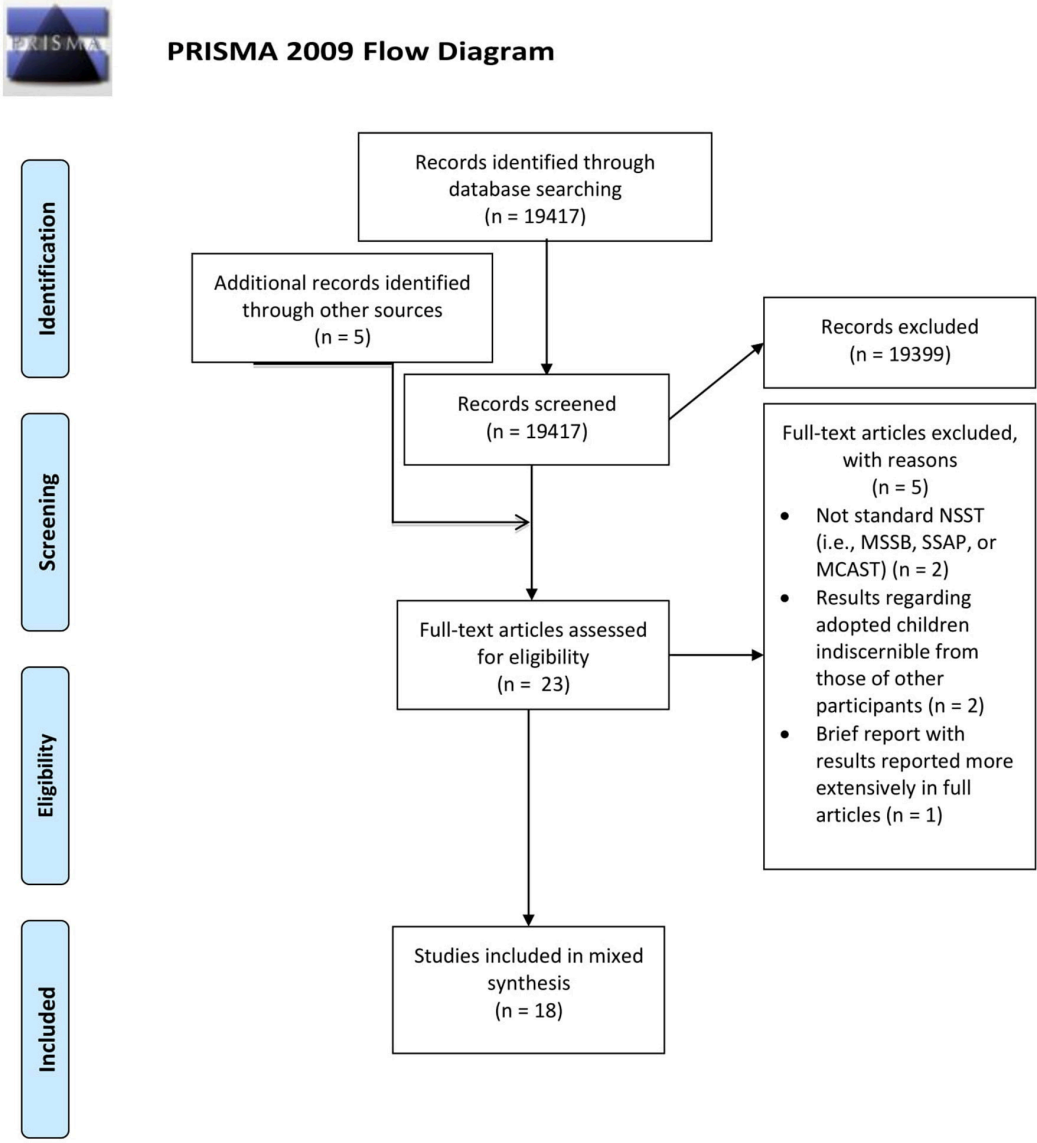
PRISMA flowchart. Figure reproduced with permission under the Creative Commons Attribution License from: Moher et al. (2009).

A systematic literature search was conducted in May 2016 through the electronic databases *ScienceDirect, Wiley Online Library, Ovid (including PsycArticles), Taylor & Francis Online*, and *Sage*. Based on the eligibility criteria, the search strings [(“adopted child” OR adoptee) AND narratives] and [(“adopted child” OR adoptee) AND “story stems”] were used. After a first screening of titles and abstracts, 18 records were selected. All full-text papers were screened for eligibility, resulting in five records being excluded. Reference searching of the remaining 13 records yielded an additional five papers being identified, resulting in 18 records being included in the present review, comprising six primary qualitative, 10 primary quantitative, and two primary mixed-methods studies. These 18 records report on seven unique studies (see information in Table 1 for which records report on the same study). The PRISMA flowchart is presented in Figure 1, and characteristics of the included studies are presented in Table 1.

**Table 1:** Characteristics of studies included in review.

**Reference**	**Adoption subsample**	**Comparison group(s)**	**Assessment times and measures**	**Research type**	**Theoretical perspective**
Barone and Lionetti, 2012	*N* = 20 (age range = 3–5 y, *M* age = 3.9 y, *SD* = 1.4; 16 boys) and their adoptive mothers (*M* age = 39.9 y, *SD* = 4.2) and fathers (*M* age = 41.9 y, *SD* = 3.5)	N/A	12–18 mos after adoption: MCAST 12 mos after MCAST: TEC Within first mo of adoption: AAI	Quan	Att – Cat
Heller et al., 2006^a^	Fraternal twins aged 8, boy and girl	N/A	NSST largely based on ASCT and MSSB	Qual	Att – Fine
Hodges and Steele, 2000^b^	Multiple case examples, mostly from boy aged 7	N/A	SSAP	Qual	Att – Fine
Hodges et al., 2003^b^	*N* = 33 late-adopted (placed between 4 and 8 y 8 mos of age, *M* age = 6 y 1 mo; 14 boys and 19 girls)	31 early-adopted (placed below 12 mos of age, *M* age = 3.73 mos; 15 boys and 16 girls)	Immediately after adoption (*M* age = 6 y 5 mos for late-adopted group; 5 y 9 mos for early-adopted group) and 1-y follow-up: SSAP	Quan	Att – Fine
Hodges et al., 2005^b^	*N* = 63 late-adopted (placed between 4 and 8 y 8 mos of age, *M* age = 6 y; just over half were boys)	48 early-adopted (placed below 12 mos of age, *M* age = 3.73 mos; half were boys)	Immediately after adoption (*M* age = 6 y 4 mos for late-adopted group; 5 y 9 mos for early-adopted group), 1- and 2-y follow-up: SSAP Immediately after adoption: SDQ	Quan	Att – Dim/ Fine
Hodges et al., 2009^b^	Multiple case examples (girl aged 7, girl aged 5.5, boy aged 8, boy aged 7)	N/A	SSAP	Qual	Att – Dim/ Fine
Kocovska et al., 2012	*N* = 34 referred with symptoms of indiscriminate friendliness and history of severe maltreatment in early childhood (age range = 5–12 y, *M* age = 9.4 y, *SD* = 1.8; 18 boys and 16 girls)	32 typically developing age- and gender-matched comparisons with no history of maltreatment (*M* age = 8.7 y, *SD* = 2.4; 17 boys and 15 girls)	MCAST at *M* of 51.3 (*SD* = 26.8) mos in adoptive family for referred group	Quan	Att – Cat
Pace et al., 2014^c^	*N* = 61 late-placed (age range = 4.5–8.3 y, *M* age = 6.2 y, *SD* = 1.1; 34 boys and 27 girls)	N/A	MCAST at *M* of 13.7 mos (*SD* = 6.6, range = 7-28) after placement	Quan	Att – Cat/ Dim Mz
Pace and Zavattini, 2011^c^	*N* = 20 late-placed (age range = 4–7 y, *M* age = 71.7 mos, *SD* = 12.4; 9 boys and 11 girls) and their (15) adoptive mothers (*M* age = 44.1 y, *SD* = 4.1)	N/A	6 mos after placement: MCAST At placement: AAI	Quan	Att – Cat
Pace et al., 2012^c^	*N* = 28 late-placed (age range = 4-7 y, *M* age = 70.0 mos, *SD* = 12.8; 13 boys and 15 girls) and their (20) adoptive mothers (age range = 38–52 y, *M* age = 44.5 y, *SD* = 4.4)	N/A	7-8 mos after placement: MCAST 40 days after placement: AAI 40 days after placement: LIPS-R 7–8 mos after placement: PPT	Quan	Att – Cat/ Fine
Page et al., 2008^a^	See (Heller et al., 2006)	See (Heller et al., 2006)	See (Heller et al., 2006)	Qual	Att – Fine Aff
Román et al., 2012	*N* = 40 (age range = 4–8 y, *M* age = 76.0 mos, *SD* = 14.2; 72.5% boys)	50 institutionalized (age range = 4–8 y, *M* age = 78 mos, *SD* = 17.9; 48% boys) and 58 biological comparisons (age range = 4–8 y, *M* age = 75 mos, *SD* = 14.6)	SSAP BDI, CEG	Quan	Att – Dim
Steele et al., 2007a^b^	See (Steele et al., 2003)	See (Steele et al., 2003)	Immediately after adoption: SSAP Immediately before adoption: AAI 3 mos after adoption: PDI	Quan	Att – Fine
Steele et al., 2003^b^	*N* = 61 late-adopted maltreated (age range = 4–8 y, *M* age = 6; 43% boys) and their (43) adoptive mothers (*M* age = 40 y)	N/A	SSAP AAI	Mix	Att – Fine
Steele et al., 2010^b^	Case example (boy aged 4.5)	N/A	At and 2 y into placement: SSAP AAI	Qual	Att – Fine Aff Mz
Steele et al., 2009^b^	Two case examples (girl aged 7 and girl aged 6.5)	N/A	Within 3 mos of and 2 y into placement: SSAP Prior to adoption: AAI	Qual	Att – Fine
Steele et al., 2008^b^	*N* = 58 late-adopted maltreated (age range = 4–8 y, *M* age = 5.5, *SD* = 1.4; 43% boys) and their (41) adoptive mothers (*M* age = 40 y, *SD* = 6) and fathers (*M* age = 43 y, *SD* = 7)	47 biological comparisons and their (32) parental couples	2 y after adoption: SSAP Prior to adoption: AAI	Mix	Att – Fine
Vorria et al., 2006	*N* = 61 (age range = 3.8–4.8 y, *M* age = 4.2; 32 boys and 29 girls)	39 comparisons (age range = 3.8–4.8y, *M* age = 4.2, *SD* = 0.2; 20 boys and 19 girls)	ASCT MSCA	Quan	Att – Fine

*References with the same superscript report on the same study*;

*N/A, Not Applicable*;

*MCAST, Manchester Child Attachment Story Task; TEC, Test of Emotion Comprehension; AAI, Adult Attachment Interview; ASCT, Attachment Story Completion Task; MSSB, MacArthur Story Stem Battery; SSAP, Story Stem Assessment Profile; SDQ, Strengths and Difficulties Questionnaire; LIPS-R, Leiter International Performance Scale – Revised; PPT, Peabody Picture Test; BDI, Battelle Development Inventory; CEG, Comprensiòn de Estructuras Gramaticales; PDI, Parent Development Interview; MSCA, McCarthy Scales of Children's Abilities*;

*Quan, primary quantitative study; Qual, primary qualitative study, Mix, primary mixed-methods study*;

*Att, attachment perspective; Cat, attachment categories; Dim, attachment dimensions; Fine, fine-grained aspects of attachment; Aff, affect-regulatory perspective; Mz, mentalizing perspective*.

### Quality assessment of studies

Assessing the quality of the primary studies was considered as an essential part of the present review, though not a priori as a reason for exclusion (Heyvaert et al., 2017). All included studies were assessed with regard to methodological quality, using the QARI Critical Appraisal Tool for “Interpretive and Critical Research” and “Observational Studies” (Joanna Briggs Institute, 2011) rated independently by two of the authors (NV and DB), followed by joint discussion (Table 2).

**Table 2:** Quality assessment of studies included in review.

**Criterion**	**JBI QARI critical appraisal tool for interpretive and critical research**					**JBI QARI critical appraisal tool for observational studies**					
1. Theoretical framework	1. Is there congruity between the stated philosophical perspective and the research methodology?					
2. Appropriate design (including sampling and sample characteristics)	2. Is there congruity between the research methodology and the research question or objectives?					1. Is the study based on a random or a pseudo-random sample? 2. Are criteria for inclusion in the sample clear? 3. If comparisons are being made, was there sufficient description of the groups?				
3. Data collection procedure	3. Is there congruity between the research methodology and the methods used to collect data?					
4. Data analysis procedure	4. Is there congruity between the research methodology and the representation and analysis of data?					4. Was an appropriate statistical analysis used?				
5. Findings	5. Is there congruity between the research methodology and the interpretation of results?					5. Were outcomes assessed using objective criteria?				
6. Context	6. Is there a statement locating the researcher culturally or theoretically?					
7. Impact of investigator	7. Is the influence of the researcher on the research, and vice versa, addressed?					
8. Believability	8. Are participants and their voices, adequately represented?					
9. Ethics	9. Is the research ethical according to current criteria or, for recent studies, is there evidence for ethical approval by an appropriate body?					
10. Outcome	10. Do the conclusions drawn in the research report flow from the analysis, or interpretation, of the data?					
**Reference**	**Research type**	**Theoretical framework**	**Appropriate design**	**Data collection procedure**	**Data analysis procedure**	**Findings**	**Context**	**Impact of investigator**	**Believability**	**Ethics**	**Outcome**
Barone and Lionetti, 2012	Quan	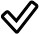	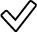	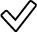	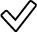	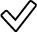	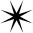	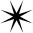	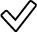	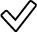	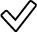
Heller et al., 2006	Qual	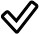	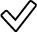	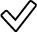	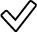	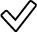	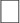	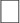	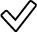	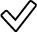	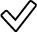
Hodges and Steele, 2000	Qual	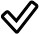	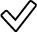	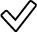	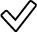	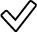	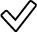	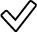	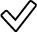	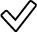	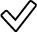
Hodges et al., 2003	Quan	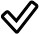	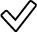	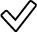	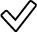	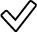	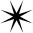	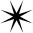	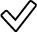	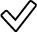	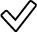
Hodges et al., 2005	Quan	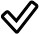	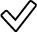	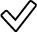	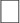	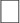	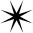	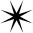	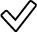	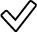	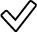
Hodges et al., 2009	Qual	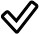	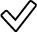	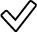	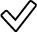	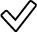	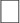	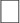	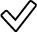	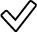	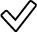
Kocovska et al., 2012	Quan	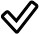	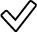	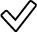	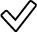	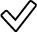	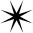	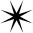	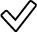	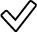	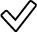
Pace et al., 2014	Quan	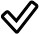	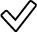	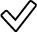	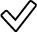	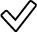	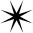	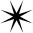	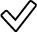	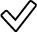	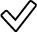
Pace and Zavattini, 2011	Quan	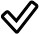	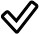	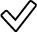	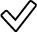	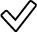	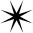	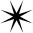	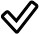	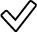	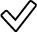
Pace et al., 2012	Quan	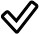	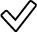	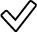	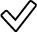	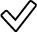	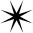	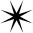	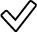	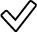	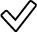
Page et al., 2008	Qual	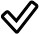	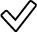	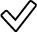	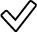	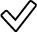	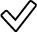	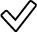	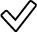	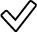	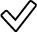
Román et al., 2012	Quan	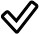	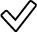	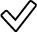	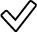	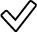	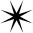	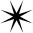	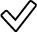	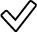	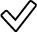
Steele et al., 2007a	Quan	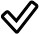	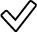	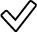	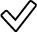	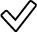	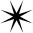	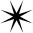	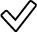	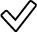	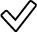
Steele et al., 2003	Mix	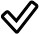	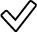	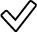	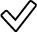	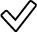	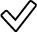	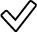	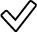	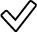	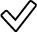
Steele et al., 2010	Qual	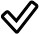	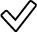	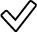	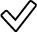	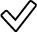	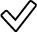	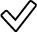	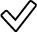	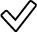	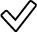
Steele et al., 2009	Qual	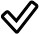	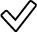	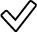	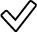	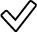	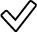	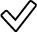	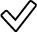	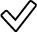	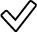
Steele et al., 2008	Mix	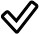	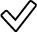	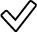	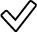	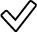	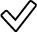	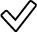	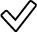	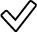	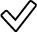
Vorria et al., 2006	Quan	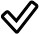	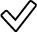	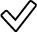	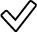	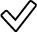	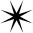	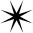	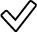	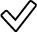	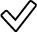

*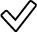, Criterion is met*;

*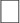, Criterion is not addressed*;

*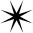, Not applicable*.

### Data extraction and synthesis

Data were extracted from the studies and synthesized using an integrated design (Sandelowski et al., 2006), that is, findings from all included studies were extracted and synthesized in an interactive dialogue in which all data from the primary studies, whether qualitative, quantitative, or mixed, were ascribed equal status and were analyzed concurrently (Heyvaert et al., 2013), using tables with emerging thematic headings. A meta-analysis was neither appropriate nor feasible (due to the small number of and large diversity in quantitative studies) for data synthesis.

## Results

As shown in Table 1, most studies to date have used NSSTs exclusively in an attachment perspective. In what follows, we first discuss why NSSTs have proven particularly useful in assessing adopted children's IWMs, paying attention to (a) the advantages of NSSTs as an assessment method compared with other methods to gain insight into IWMs, and (b) global vs. more fine-grained coding of adopted children's responses to NSSTs (section Suitability of NSSTs for Assessment of Adopted Children in Attachment Perspective). Next, we provide an overview of findings from studies (a) in which NSSTs were used as a measure of child attachment *per se* (section Child Attachment), (b) to gain insight into other child characteristics potentially influencing or influenced by child attachment as assessed by NSSTs (section Child Characteristics Potentially Influencing or Influenced by Child Attachment), and (c) investigating the role of parental attachment in developmental change in adopted children's IWMs as assessed by NSSTs (section Role of Parental Attachment in Developmental Change in Children's IWMs). We then broaden the scope to two complementary perspectives that have been suggested in the literature (section Broadening the Scope From Attachment to Affect-Regulatory and Mentalizing Perspectives). Finally, we argue for the value of NSSTs in tailoring treatment to a particular adopted child's needs (section From Assessment to Tailored Treatment).

### Suitability of NSSTs for assessment of adopted children in attachment perspective

#### NSSTs as a route to understanding IWMs

Unsurprisingly, as all reviewed studies used story stems as an attachment-based instrument to assess adopted children, all authors advocated the value of an attachment perspective in understanding these children's difficulties. NSSTs are based on a natural mode of self-expression for children, which facilitates direct assessment of children's representations in a non-intrusive way (Hodges and Steele, 2000; Hodges et al., 2003, 2005, 2009; Heller et al., 2006; Page et al., 2008). Notably, in responding to story stems, children are not directly reporting on real (past or current) family experiences, nor are they merely fantasizing (Hodges and Steele, 2000); rather, they are expressing aspects of their (in)ability to cope with and give meaning to these experiences (Hodges et al., 2005, 2009). The latter also implies that, across an array of different story stem completions, NSSTs provide a “window” into a child's ability to imagine what can happen in the hypothetical situations depicted in the different stems (Hodges et al., 2009).

Hodges and Steele (2000) and Hodges et al. (2005) refer to two major advantages of the use of story stems over self-report measures. First, play can reveal aspects of *procedural memory*, that is, experiences in the child's life that are not part of verbally based memory but are recalled in perceptual, affective, or physical (sensorimotor) form. Procedural memory operates automatically, outside conscious awareness, and therefore requires assessment by means other than conscious verbal recall or description (Hodges and Steele, 2000; Hodges et al., 2003, 2005), such as play (Steele et al., 2003, 2010). Children who have experienced early adversity may give behavioral evidence of their fearful or aversive expectations of their parents, without necessarily being able to recall specific events (Hodges et al., 2005). Furthermore, the opportunity to play provides an intermediate space in which the child can show his/her ability to cope with relational content that is too painful or anxiety-provoking, for instance, by avoiding or transforming the core dilemma of the story stem, “revealing some of the defenses employed to make thinking about it bearable” (Hodges and Steele, 2000, p. 435).

A second advantage of story stems in the traditions of the MacArthur Story Stem Battery (Bretherton et al., 1990) and the Attachment Story Completion Task (Bretherton et al., 1990), such as the Story Stem Assessment Profile (Hodges et al., 2007), lies in the psychological distancing provided through *displacement*. Children are asked about a standard doll family rather than trying to replicate their own family configuration (Hodges and Steele, 2000; Hodges et al., 2003, 2005; Page et al., 2008). Some of the story stems use animal figures, providing further displacement from the child's personal experience. As children with early adversity sometimes seem to experience even dilemmas portrayed with doll figures as too “close to the bone” and anxiety-provoking (Hodges et al., 2003, 2005), attributing difficult emotions or behaviors to animal figures may facilitate the child's narrative expression. In this context, it is important to note that in the Manchester Child Attachment Story Task (MCAST; Green et al., 2000) the child's identification with the doll figures is explicitly emphasized by asking the child to choose dolls to represent him/herself and his/her caregiver.

Despite these advantages, there is a large variety across studies in how story stem batteries are composed and in how children's responses to the story stems are coded, rendering direct comparison between study results difficult^2^. However, even in view of the variety of coding procedures, two approaches are discernible on the whole.

#### Global vs. more fine-grained perspectives on IWMs

In assessing the attachment representations of adopted children, two different approaches are used. Some studies look at children's global attachment style in a categorical or dimensional way. Others view story stem responses from a more detailed and fine-grained perspective, assessing the component elements subsumed under the general construct of a “secure” or other attachment organization (Hodges et al., 2005, 2009). These latter authors argue that it is not particularly useful merely to establish the child's global attachment style, as children who have experienced early adversity are very unlikely to show “secure” attachment organization and are likely to be “disorganized” (Carlson et al., 1989; Hodges et al., 2005, 2009). Similarly, in examining changes in attachment during placement, to expect to find that a child shifts from an “insecure” to a “secure” category of attachment organization would be too gross a categorization to be useful (Hodges et al., 2005). Developmental recovery in these children can take a long time, and it is likely that some of the effects of their earlier experiences will remain with them permanently or at least well into adulthood (Howe, 1998; Hodges et al., 2005).

Fourteen records included in this review reported on the findings of six unique studies (a) in which NSSTs were used as a measure of child attachment *per se* (section Child Attachment), (b) to gain insight into other child characteristics potentially influencing or influenced by child attachment as assessed by NSSTs (section Child Characteristics Potentially Influencing or Influenced by Child Attachment), and (c) investigating the role of parental attachment in developmental change in adopted children's IWMs as assessed by NSSTs (section Role of Parental Attachment in Developmental Change in Children's IWMs). All but four of these 14 papers reported on quantitative findings: the Steele et al. (2009, 2010) papers adopted a qualitative approach, whereas the Steele et al. (2003, 2008) papers adopted a mixed-methods approach.

### Child attachment

#### Attachment classifications

Three studies, reported in five papers (Pace and Zavattini, 2011; Barone and Lionetti, 2012; Kocovska et al., 2012; Pace et al., 2012, 2014), investigated the distribution of attachment classifications in adopted children (Table 3). Across all three studies, the prevalence of children classified as insecurely attached ranged from 44 to 75%. Of clinical importance is the prevalence of children classified as disorganized, estimated to range between 32 and 37%. These findings are in line with the results of a meta-analysis of attachment in adopted children (van den Dries et al., 2009) showing that 47% of children were securely attached and 31% were disorganized. Importantly, the percentage of adopted children with disorganized attachment is significantly higher than the percentage of disorganized children in normative samples (e.g., Barone et al., 2009; for a meta-analysis, see van Ijzendoorn et al., 1999) and comparable to the percentage of disorganized children growing up in high-risk circumstances as reported by van Ijzendoorn et al. (1999) and Gloger-Tippelt and Kappler (2016).

**Table 3:** Distribution of attachment classifications.

	**Reference**	**Sample characteristics**	**Attachment measure**	**Secure (%)**	**Global insecure^3^ (%)**	**Insecure avoidant (%)**	**Insecure ambivalent (%)**	**Disorganized (%)**
Adoptive sample	Barone and Lionetti, 2012	*N* = 20, Italian study, *M* = 3.9 y, *SD* = 1.4	MCAST	25	75	30	10	35
	Kocovska et al., 2012	*N* = 34, United Kingdom study, *M* = 9.4 y, *SD* = 1.8	MCAST	56	44			32
	Pace et al., 2014,^4^	*N* = 61, Italian study, *M* = 6.2 y, *SD* = 1.1	MCAST	47	53	15	2	37
	van den Dries et al., 2009	Meta-analysis in adoptive samples (17 studies, *N* = 468, age range = 0–12 y)	SSP or AQS	47				31
Normative sample	Barone et al. (2009), comparison group reported in Barone and Lionetti (2012)	*N* = 230, Italian study, *M* = 6.7 y, *SD* = 1.2	MCAST	63	37	16	10	11
	Gloger-Tippelt and Kappler, 2016	Pooled analyses of 14 non-risk samples in Germany (*N* = 642)	GASCP	36.6	63.4	36.8	15	11.6
		Pooled analyses of 8 risk samples in Germany (*N* = 245)	GASCP	25.3	74.7	33.5	8.6	32.7
	van Ijzendoorn et al., 1999	Meta-analysis in normal, middle class, nonclinical groups in North America (*N* = 3,141)	Not reported	62	38	15	9	15
		Meta-analysis in low SES samples (*N* = 586)	Not reported					25
		Meta-analysis in maltreating parents (*N* = 165)	Not reported					48–77

*MCAST, Manchester Child Attachment Story Task; SSP, Strange Situation Procedure; AQS, Attachment Q-Sort; GASCP, German Attachment Story Completion Procedure*.

#### Attachment dimensions

Román et al. (2012) found that both adopted and institutionalized children displayed more indicators of insecurity, avoidance, and disorganization than the control group (children living with their birth families, with no history of maltreatment). These findings are only partially in line with the meta-analytic results of van den Dries et al. (2009), reporting that adopted children showed less disorganization than institutionalized children, but more disorganization than non-adopted biological peers. One possible explanation for this discrepancy is that the adopted children studied by Román et al. (2012) spent on average 2 years in institutional care, rendering the differentiation between adopted and institutionalized children less clear.

Pace et al. (2014) found that children classified as securely attached received higher scores on coherence of mind (i.e., narrative coherence, as expressed in quality, quantity, relevance, and manner) compared with children classified as insecurely attached. Hodges et al. (2005) investigated how the attachment dimensions of adopted children changed over time. Two years after adoption, the children showed a decrease in global defense/avoidance scores. However, indicators of attachment disorganization in their stories remained largely unchanged over 2 years.

#### Fine-grained aspects of attachment

Vorria et al. (2006) concluded that adopted children showed lower scores on story resolution, narrative coherence, and prosocial themes, and a higher score on avoidance, compared with a group of biological children, even after adjusting for children's cognitive developmental level. However, the adopted and comparison groups did not differ significantly with regard to the occurrence of atypical and negative themes.

Hodges et al. (2003, 2005) investigated how fine-grained aspects of attachment evolved in the first 2 years after adoption (Table 4). Overall, aspects of new and more positive representations had begun to develop; however, already-established negative representations persisted. Using a qualitative approach, Steele et al. (2010) illustrated the former with a case study of “Larry,” a boy aged 4.5 years at the time he was first placed with his adoptive parent. At this time he was at first unwilling to acknowledge the distress as outlined in the story stem, followed by increasingly aggressive and catastrophic content instead of helping parental representations. Two years later, this boy's completion of the same story stem was characterized by helping parental representations and realistic/pleasurable domestic life.

**Table 4:** Longitudinal fine-grained attachment results from Hodges et al. (2003) and Hodges et al. (2005).

	**1-year follow-up (Hodges et al.**, **2003****)**		**2-year follow-up (Hodges et al., 2005)**

	**Changed**	**Unchanged (level)**	**Changed**
Regulation strategies	- Less avoidance	- Disorganization (high) - Extreme aggression (high)	- Acknowledge more distress in adults and children
Adult representations	- More adults helping - More adults setting limits - Less adult unaware	- Adults showing affection - Adults as aggressive, rejecting, unaware of children's needs, injured or dead (high)	- More adults providing practical help, emotional comfort and affection - More adults setting limits and punishing physically, without the child having to resort to avoidance or being overwhelmed with imagery or extreme violence and death - Adults more aware of children's need for help and comfort - More secure sense that attachment needs would be met, that help and comfort would be available, and adults would know when they are needed
Child representations	- More children helping - More realistic mastery - More children seeking help - Less turning on self	- Child endangered or injured/dead	- More children seeking help - More children helping other children
Positive adaptation	- Less use of magic/omnipotence	- Representations of domestic life (low)	- Domestic life represented in a more realistic and positive way - Less use of magic/ omnipotence

### Child characteristics potentially influencing or influenced by child attachment

Five studies, reported in seven papers (Hodges et al., 2003, 2005; Vorria et al., 2006; Barone and Lionetti, 2012; Pace et al., 2012, 2014; Román et al., 2012), investigated one or more factors influencing or influenced by child attachment: (a) child gender, (b) characteristics of the adoption process, (c) developmental covariates, and (d) developmental outcomes.

#### Child gender

Pace et al. (2012) reported that the distribution of attachment categories did not differ significantly between boys and girls. However, in a follow-up study, Pace et al. (2014) found that, from a categorical perspective, boys were rated significantly more insecure and disorganized than girls; from a dimensional approach, they showed significantly higher disorganization and a trend for lower coherence of mind scores compared with girls. In the same vein, Román et al. (2012), using attachment dimensions, found that girls showed more security and less insecurity than boys.

#### Characteristics of the adoption process

Pace et al. (2012) found that the distribution of attachment categories did not differ significantly between *domestically and internationally adopted* children. Similarly, Pace et al. (2012, 2014) reported that the presence/absence of *siblings* in the family had no significant influence on the distribution of attachment categories. However, Román et al. (2012), comparing adoption of single children vs. siblings, found that although there were no differences in security and avoidance, children adopted with a sibling showed fewer signs of insecurity and disorganization.

Pace et al. (2012, 2014) found that the distribution of attachment categories did not differ significantly by *age at adoption*. Similarly, Román et al. (2012) failed to find significant correlations between age at adoption and attachment dimensions. However, Pace et al. (2014), surprisingly, found a significant negative correlation between age at adoption and a dimensional disorganization score (but not coherence of mind or mentalizing skills). Adopting a more fine-grained perspective on attachment, Hodges et al. (2003, 2005) found meaningful differences between early- and late-adopted children (see Table 5). Interestingly, when comparing the effects of age at placement at different time-points, Hodges et al. (2005) found that, overall, positive changes in attachment aspects were larger and took place more quickly in younger vs. older children. Late-adopted children's display of many characteristics of disorganization remained unchanged over 2 years, suggesting that they were still struggling with disorganizing emotional responses, but the reduction in avoidance suggests that they had become more able to represent these in the narrative, acknowledging dilemmas and maintaining better functioning in the face of stress.

**Table 5:** Significant and non-significant fine-grained attachment differences between early- and late-adopted children.

	**Immediately after adoption (Hodges et al.**, **2003****)**		**1-year follow-up (Hodges et al.**, **2005****)**	

	**Significant**	**Non-significant**	**Significant**	**Non-significant**
Regulation strategies	Compared to early-adopted children, late-adopted children: - Tended to be less engaged in/ more avoidant of story completion, particular dilemmas or constraints within the story - Showed significantly higher levels of catastrophic fantasy and bizarre/atypical answers (but not bad-good shifts) in their stories, which are considered indicators of disorganization - Showed significantly higher levels of extreme aggression (but not coherent aggression) in their stories		- Compared to late-adopted children, early-adopted children exhibited a larger decrease in aspects of avoidance	- A decrease in global defense/avoidance scores - Indicators of attachment disorganization remained unchanged
Adult representations	Compared to early-adopted children, late-adopted children: - Were significantly less likely to show adults helping children and adults showing affection - Were significantly more likely to show adults as aggressive, rejecting, unaware of their needs, and injured/dead in their narratives - Scored lower on representations of physical punishment by parents, which was a somewhat surprising finding	- Representations of parental limit setting	Compared to late-adopted children, early-adopted children: - Exhibited a larger increase in positive representations - Still scored higher on representations of physical punishment	
Child representations	Compared to early-adopted children, late-adopted children: - Were less likely to show children helping - Showed less realistic mastery - Were more likely to show children as injured/dead	- Child seeking help - Child endangered - “Turning on self”		
Positive adaptation	- Compared to early-adopted children, late-adopted children were less likely to include domestic life in their stories	- Level of magic/omnipotence.		

Furthermore, Pace et al. (2012, 2014) found that the distribution of attachment categories did not differ significantly by *duration of placement* (but Pace et al., 2014 did report that longer-placed children showed higher mentalizing scores as assessed by NSST). Interestingly, more detailed analyses by Román et al. (2012) indicated that although age at adoption did not influence dimensional attachment scores, when this variable was entered simultaneously with “time in adoptive family” in a hierarchical regression, both predictors were positively related to security scores in adopted children. This suggests that time spent in the adoptive family is an important aspect for developmental catch-up with regard to attachment. Moreover, Román et al. (2012) reported that having a significant family experience prior to adoption was associated with lower avoidance than having spent the pre-adoption time in institutions. In the subsample of adopted children with family experience before adoption, the amount of time spent with a family before institutionalization was positively related to security. In the same vein, older age at entrance to an institution and shorter experience there were related to higher security and lower insecurity. However, in the subgroup of adopted children who had spent their whole pre-adoption life in an orphanage, none of these variables showed significant relationships with attachment representations.

#### Developmental covariates

Pace et al. (2012) found no significant correlations between attachment categories and the child's level of schooling, non-verbal IQ, or language comprehension. In a follow-up study, Pace et al. (2014) again found no associations between secure/insecure classifications and children's level of schooling (or maternal age or level of education). The findings reported by Román et al. (2012), using attachment dimensions, are more mixed. These authors found that although linguistic competence did not influence attachment indicators, children with higher intelligence showed more indicators of secure attachment. In line with these latter findings and from a fine-grained perspective, Vorria et al. (2006) found a positive association between cognitive capacities and story resolution, narrative coherence, and prosocial themes, and a negative association between cognitive capacities and avoidance.

#### Developmental outcomes

Barone and Lionetti (2012) found that adopted children classified as disorganized performed worse on emotional competence compared with adopted children with coherent attachment organizations, regardless of security, whereas no difference was found between securely and non-securely attached children. However, Pace et al. (2014) found that children classified as secure received higher scores on mentalizing skills than children classified as insecure. From a fine-grained perspective, Hodges et al. (2005) found that children with more conduct problems showed more adult aggression, catastrophes, denial or distress, and avoidance in their story completions, and less realistic mastery. Furthermore, children with more peer problems showed more denial of distress and catastrophes, and less realistic mastery, as well as fewer instances of children helping other children. In contrast, children with more prosocial behavior showed more realistic mastery, acknowledgment of distress, children helping other children, and realistic/pleasurable domestic life, and less avoidance.

### Role of parental attachment in developmental change in children's IWMs

Quantitative and qualitative studies have shown that adoptive parents' attachment representations play an important role in determining the newly formed relationship with their adopted child across time (Steele et al., 2003, 2008, 2009, 2010).

#### Attachment classifications

Two studies, reported in three papers (Pace and Zavattini, 2011; Barone and Lionetti, 2012; Pace et al., 2012), investigated the concordance between children's attachment categories, assessed 6–18 months after placement, and their adoptive mothers' attachment as indexed by Adult Attachment Interview (AAI; George et al., 1985) categories around the time of placement. Barone and Lionetti (2012) found a significant concordance between children's and mothers' attachment of 60% using a three-way classification (secure vs. insecure-avoidant vs. insecure-ambivalent) and 80% using a two-way classification (secure vs. insecure). No significant concordance was found between children's and fathers' attachment. However, the authors reported that all children classified as secure had at least one parent with a secure attachment pattern, and 75% of these children had two parents with secure attachment. In contrast, Pace and Zavattini (2011) and Pace et al. (2012) did not find a significant concordance between children's and mothers' attachment using a two-way classification. Specifically, Pace et al. (2012) reported that 40% of disorganized children were adopted by mothers with a dismissing state of mind, and 10% by mothers with an unresolved state of mind.

#### Fine-grained aspects of attachment

Two studies, reported in six papers (Steele et al., 2003, 2007a, 2008, 2009, 2010; Pace et al., 2012), tapped into more fine-grained aspects of children's attachment and associations with parental attachment. Pace et al. (2012), in addition to—and, importantly, in contrast to—the categorical approach, found significant associations between maternal AAI states of mind, assessed 40 days after placement, and MCAST global scales, administered 7–8 months after placement. Specifically, maternal “Idealization of mother” was significantly negatively related to children's “Coherence of mind.” In addition, although both maternal “Idealization of mother” and “Anger toward father” were significantly positively related to children's “Global disorganization score,” only the former accounted for unique variance when entered simultaneously as predictors of children's disorganization.

Steele et al. (2003), using another fine-grained coding scheme, found that the story completions of children, gathered immediately after adoption, adopted by “insecure” mothers (as opposed to “secure” mothers; as assessed immediately before adoption) were more likely to show (a) catastrophic fantasies; (b) child aggression; (c) adult aggression; (d) throwing out or throwing away; (e) bizarre or atypical content; (f) child injured/dead; and (g) adult injured/dead. Subsequently, Steele et al. (2003, 2007a) calculated a composite aggressiveness score, “child aggression,” based on these seven themes^5^. In general, the “child aggression” composite was found to be related to maternal attachment (assessed by the AAI) and parents' representations of their children (assessed by the Parent Development Interview; Aber et al., 1985; 3 months into placement). Detailed findings are presented in Table 6.

**Table 6:** Associations between parental attachment and fine-grained child attachment aspects.

	**Steele et al., 2003**	**Steele et al., 2007a**
Child aggression composite	- Significantly positively associated with mothers' insistence on the inability to recall their childhood (a linguistic feature indicative of an insecure-dismissing state of mind), as well as with mothers' derogation of their own fathers - Significantly negatively related to mothers' coherence of mind and coherence of transcript (which can be considered hallmarks of an autonomous-secure state of mind)	- Positively correlated with Insecurity in the maternal AAIs - Significantly negatively correlated with “positive/reflective” dimension of the PDI (i.e., parent's global reflectiveness on the relationship, the overall richness of perception of the child, global coherence, parent's level of child focus, parental warmth, and parental joy/pleasure) - Significantly positively related to the “despair/lack of satisfaction” dimension of the PDI (i.e., parental need for social support, lack of satisfaction with support received, parental disappointment) - Not predicted by AAI autonomy/security and PDI positivity/reflectivity when these were entered simultaneously as predictors - Marginally significantly predicted by PDI despair/lack of satisfaction when controlling for AAI autonomy/security (the latter was rendered non-significant)
Other findings	- Children adopted by “unresolved” mothers as opposed to “not unresolved” mothers (i.e., resolved or lacking past experiences of loss or trauma) were more likely to show (a) higher scores for parent appearing childlike, (b) adult aggression, and (c) throwing out or throwing away, and (d) lower scores for realistic mastery and (e) sibling or peer helps	- AAI unresolved mourning and PDI despair/lack of satisfaction accounted for unique variance in “placing parent in a childlike position”

Steele et al. (2008) calculated two composite scores: a “disorganization composite,” comprising (a) catastrophic fantasies; (b) bizarre/atypical material; (c) bad-to-good shifts; (d) extreme aggression; (e) magic omnipotence; and (f) child appearing parent-like or role reversal; and an “insecure composite,” comprising (a) child endangered; (b) child injured/dead; (c) adult unaware; (d) adult actively rejecting; (e) adult injured/dead; (f) excessive compliance; (g) extreme aggression; (h) neutralization; and (i) throwing away. Subsequently, for both composites, the sample was divided into three equal groups of children with “low,” “medium,” and “high” scores. Steele et al. (2008) reported that when one or both parents were securely attached, as assessed by the AAI at the time of adoptive placement, children were less likely to belong to the high insecure or disorganized group 2 years later. By contrast, when neither parent's AAI was secure (i.e., Insecure—either Dismissing or Preoccupied), 86% of the children scored in the highest group for disorganization.

Finally, in a qualitative approach, Steele et al. (2009) presented two cases by means of the mothers' pre-adoptive AAI transcript excerpts and their children's story stem completion examples to illustrate their findings: one in which the mother's AAI was classified as autonomous-secure and one in which the mother was classified as insecure-dismissing. Although both children showed indices of Insecurity in their story stem completions early in the placement, at 2-year follow-up, the story stem completions of the adopted child placed with the secure parent showed many themes indicative of Security, whereas those of the adopted child placed with the insecure-dismissing parent continued to show a preponderance of themes indicative of Insecurity, Disorganization, and Defensive Avoidance (Steele et al., 2009). In this regard, Steele et al. (2010) hypothesized about what it is that parents with secure-autonomous states of mind do to facilitate these positive changes in their adopted children. First, they stated that parents' secure-autonomous states of mind might be associated with open and flexible processing of affect, which in turn helps the child to modulate negative affect. Furthermore, Steele et al. (2010) proposed that in parents with secure-autonomous states of mind complementary processes are at play, involving awareness of their own and the child's mental states, and the ability to envision a way forward that brings parent and child together. This stance nurtures a similar ability for the child to integrate a range of feeling states because the new parental environment supports exploration and integrative efforts. Although, to the best of our knowledge, both perspectives—affect-regulatory and mentalizing—have not been thoroughly tested empirically to date, Steele et al.'s (2010) theorizing sets the stage for broadening the scope on the use and usefulness of NSSTs.

### Broadening the scope from attachment to affect-regulatory and mentalizing perspectives

#### Affect-regulatory perspective: from considering challenging situations to experiencing and dealing with challenging emotions

As explained above, story stems consist of mildly stressful scenarios that are part of everyday childhood experience (Steele et al., 2003; Page et al., 2008). As such, story stems not only tap into children's attachment representations by evoking something about the *content* of the child's perceptions and experiences of relationships with significant others. Story stems also immediately evoke the affects and the coping and defensive strategies children have available to manage emotionally challenging situations. As such, a child's story stem responses also shows us something about his/her *way* of dealing with emotionally challenging situations, such as how s/he modulates anxiety and aggression, attempts to master conflict, and makes defensive maneuvers (e.g., by disengaging from the task, or changing the constraints of the story; Steele et al., 2010). In this regard, Steele et al. (2010) point to the given that the IWM concept, and its meaning-making capacities, has been extended from a mere representational level toward a set of rules for how to interpret emotions and engage in behavioral strategies for managing them. Perhaps the most important function of IWMs is to regulate the individual's experience of intense emotion and to direct the individual's behavioral and psychological responses. In the same vein, Román et al. (2012) emphasize how inner representations serve as a guide for perceiving oneself and others, as well as interpreting one's emotions and regulating one's emotional behavior. In sum, the representations make up the initial content of IWMs, informing one's sense of self, others, and what to expect, but also immediately evoke affect-regulation and behavioral strategies, particularly when distressed—that is, when the attachment system is activated.

#### Adopting a mentalizing perspective

In addition to the attachment and affect-regulatory perspectives, a third perspective on NSSTs, that of mentalizing (Fonagy et al., 2002), has recently been suggested (Steele et al., 2010; Pace et al., 2014). Mentalizing refers to the capacity to understand one's own and others' behaviors as motivated from an internal world (feelings, thoughts, desires, anxieties, etc.) and the ability to guide one's actions accordingly. In “good enough” circumstances, that is, an environment that is relatively free of obstacles, the process of developing mentalizing capacities is part of ordinary parent–child interactions. Fonagy and Allison (2012) describe how secure attachment relationships ensure normal family discourse and playful interactions offering the necessary opportunities to learn about the links between internal states and actions that scaffold the development of mentalizing.

However, early adversity, as is experienced by many adopted children, interferes with the development of mentalizing capacities in two possible ways. First, these children have suffered at least one discontinuity in their early attachment relationships, and thus have had less consistent scaffolding opportunities regarding mentalizing. Second, in cases of cumulative adverse experiences (e.g., abuse or neglect) the attachment system becomes disorganized (for a comprehensive review, see Cicchetti and Valentino, 2006) and mentalizing capacities remain underdeveloped (Fonagy and Luyten, 2009) or become conflicted as a form of decoupling, inhibition, or even a phobic reaction to mentalizing (Pace et al., 2014). In the context of adversity, caregivers will be inaccurate in their reflections of the child's state of mind and the behavior of the caregiver will evoke a fear response in the child, increasing the risk of the child developing a disorganized representation of self and interfering with the capacity to reflect on mental states. For these children, to explore and think about the mind of frightening/frightened, and therefore hostile and harmful, parents would be extremely dangerous, so they block their ability to reflect, implicitly assume malignity in other people's actions, and apply this perspective to new relationships (Pace et al., 2014). Hence, such children frequently show contradictory behaviors toward their adoptive parents, oscillating between compliance, dependence, passivity, withdrawal, rejection, hostility, and provocation (Steele et al., 2010). These behaviors can make it hard to build an attachment relationship based on trust and a sense of belonging and protection in their adoptive families (Slade, 2007; Steele et al., 2007b).

Consequently, adoptive parents have to be equipped with “high quality adoptive parenting” (Pace et al., 2012, p. 47) capabilities. In this context, as discussed above, research to date has emphasized the importance of a secure attachment state of mind in parents for managing the conflicting and vulnerable representations that adopted children bring with them into their new families (Levy and Orlans, 2003; Schofield and Beek, 2005). Interestingly, “high quality adoptive parenting” may also be understood within the mentalizing framework as parents who are highly reflective and can rely on their capacity to think about their own and their child's internal reality (Sharp et al., 2006; Steele et al., 2010; Pace et al., 2014). Little research to date has specifically investigated this mentalizing perspective in adopted children using story stems. One exception is the study by Pace et al. (2014), in which MCAST story stem completions were also coded for mentalizing skills.

### From assessment to tailored treatment

As discussed earlier, NSSTs, in particular more fine-grained approaches, have proven valuable (attachment perspective) or at least promising (affect-regulatory and mentalizing perspectives) in systematically assessing adopted children's internal reality from multiple perspectives (Hodges et al., 2005; Heller et al., 2006). These techniques may also be helpfully used to examine the effects following major change in the child's external situation (Hodges et al., 2005; Heller et al., 2006). Such changes, besides placement changes, can include therapeutic intervention with a parent and also therapeutic help for children themselves (Hodges et al., 2005). Importantly, this implies that NSSTs allow us to map areas of difficulty so that parents and professionals have a clearer idea of the child's needs and vulnerabilities (Hodges et al., 2005; Pace et al., 2014).

Hodges et al. (2009), for instance, used “instrumental case studies” (Stake, 2005) to illustrate that previously maltreated children can show diverse story completions to the same story stem, reflecting the way the child has adapted to the specific characteristics of the early childhood environment and is able or unable to make use of his/her new environment. More compellingly, Page et al. (2008), by means of a fraternal twin case study (already reported on in Heller et al., 2006) described how both children, despite their common history of severe maltreatment and multiple placements, showed distinct profiles of developmental vulnerabilities and strengths based on their story stem responses; the authors argued how such individual profiles can aid in informing individually tailored therapeutic intervention. In sum, with such detailed individual understanding of the child's internal reality, parents and professionals can more sensitively tailor their parenting and their support to fit children's individual needs, thus assisting the children's developmental recovery (Hodges et al., 2005, 2009; Hodges, 2007; Page et al., 2008).

## Discussion

The findings of this narrative mixed-methods review of 18 papers in which NSSTs were used to gain insight into aspects of adopted children's (aged 3–11) internal reality may be summarized as follows. The review provides some preliminary conclusions about the suitability and value of NSSTs, as well as points to several promising avenues for research and clinical application of NSSTs. However, the small number of studies as well as the large differences in methodology among these studies need to be taken into account when discussing the implications of this body of literature carefully and critically. Of special note in this regard are: (a) different theoretical perspectives and a trend toward a shifting paradigm, rendering (b) NSSTs not merely valuable as a content-based but also as a performance-based measure; (c) the value of NSSTs not only from a disorder-oriented but also from a person-centered perspective in diagnosing and assessing adopted children's attachment vulnerabilities and changes therein; (d) preliminary findings with regard to factors influencing adopted children's attachment representations as assessed by NSSTs. (e) We formulate avenues for future research which may aid in building a more consistent body of knowledge in this domain, which may, in turn, inform clinical practice. (f) Finally, we discuss limitations of the present review.

### Toward a shifting paradigm in theoretical perspectives on NSSTs

Most studies using NSSTs with adopted children to date have used these techniques exclusively as attachment-based instruments to gain insight into adopted children's IWMs. In this regard, NSSTs have proven particularly valuable in an age group for which few self-report attachment measures are available (Bosmans and Kerns, 2015), as the opportunity to play allows children to more easily express non-conscious aspects of their internal reality stored in procedural memory, and the possibility of displacement allows children to more easily express content that would otherwise be too anxiety-provoking. The latter advantage is, however, not claimed by the developers and users of the MCAST (Green et al., 2000). In this context, it is important to note that the paucity of studies using NSSTs with adopted children, and the fact that in two of the three studies (reported in five papers) using the MCAST children's narrative responses were coded exclusively to investigate attachment classifications^6^, preclude any firm conclusions as to the importance of displacement. However, based on our own clinical experience as well as others' (Hodges and Steele, 2000; Hodges et al., 2005; Davies, 2011), we strongly advocate allowing children, particularly children who have experienced early adversity, the opportunity to approach potentially anxiety-provoking material indirectly, for instance, through the use of displacement.

The finding that all but a few of the reviewed studies used NSSTs to exclusively assess adopted children's attachment representations is striking, as several authors have suggested that both an affect-regulatory and a mentalizing perspective on NSST responses hold promise in making sense of adopted children's internal reality. To date, only one study (Pace et al., 2014) has explicitly extended the use of NSST to a mentalizing perspective.

### NSSTs as content-based as well as performance-based measures

The discussion about theoretical perspectives as described above goes hand in hand with different, though potentially complementary, perspectives on what children's NSST responses reveal precisely. Most authors emphasize that NSSTs are based on a natural mode of self-expression for children, facilitating direct assessment of representations in a non-intrusive way; whereas some point to the way affect and affect-regulation strategies and mentalizing processes can be shown in a here-and-now playful context. This raises the question whether children's story stem responses “merely” offer the clinician a window into the content of a child's internal reality and his/her expectations of caregiving others, and thus function as a projective technique; or whether they also bring affect, affect-regulation, and mentalization processes into action by bringing the child into a mildly stressful situation which s/he has to cope with here and now, and thus also function as a performance-based assessment technique. Both perspectives may be of additional value to clinical and research practice.

### NSSTs as disorder-oriented as well as person-centered perspective in diagnosing and assessing attachment vulnerabilities and changes therein

With regard to an attachment perspective, NSSTs as an assessment instrument may be of interest to researchers and clinicians alike, as children's narrative responses can be coded at three different levels—categorical, dimensional, and fine-grained—providing three complementary perspectives on the child's IWMs. In sum, the findings from the studies included in the present review reveal that despite the large variety in story stem battery composition and coding procedures and regardless of the attachment level, adopted children are at increased risk for showing signs of insecure or disorganized attachment at the time of placement. This converges with findings of studies using other attachment measures, such as the Strange Situation (e.g., Rutter et al., 2007; van den Dries et al., 2012; for a meta-analysis, see van den Dries et al., 2009), showing that adopted children exhibit more indicators of insecurity or disorganized attachment than their biological counterparts, but not more (or, even less) than their peers in institutions. These findings support the value of NSSTs in diagnosing attachment problems in adopted children.

Furthermore, findings from longitudinal studies using NSSTs suggest that, besides the relative endurance of negative IWMs, adopted children do develop more positive representations of self and other over time, which become particularly noticeable in more fine-grained aspects of attachment. Hence, an added value of NSSTs is that fine-grained analyses of a child's story stem responses can reveal precise areas of attachment-related strengths and difficulties of a particular child to a level of detail not achievable with other currently used attachment measures. From the perspective of assessing adopted children's vulnerabilities in the spectrum of attachment and changes therein, fine-grained analyses of NSST responses are particularly suitable and valuable to gain insight into these children's internal models of self and others at the time of placement, which will often be “disintegrated,” reflecting “a mind where fear and aggression often predominate” (Steele et al., 2010, p. 38), and also to track developmental change as the child settles into the adoptive family. The significance of such developmental catch-up in attachment representations is attested by the consistent finding that child attachment as assessed by NSSTs—whether operationalized categorically or in a more fine-grained way^7^—is associated with socio-emotional developmental outcomes, such as emotion comprehension, mentalizing skills, conduct and peer problems, and prosocial behavior, a finding that is in line with extant research using other attachment measures (e.g., Stams et al., 2002; Jaffari-Bimmel et al., 2006). For clinicians and researchers working with such children, it is of paramount importance that changes in aspects of a child's internal representations that take place in small steps, visible to a good observer, even long before a shift in attachment category is discernible can be mapped systematically. Such observations may be considered as windows into the child's experience that may aid the clinician in identifying opportunities to scaffold the child's representations and regulation strategies. In this context, Robinson (2016) has developed a “report format” in which the clinician can describe how the child experiences for instance, family roles or problem solving strategies, emphasizing strengths and articulating challenges; as well as subsequent recommendations on how to support the child at home as well as in treatment. It is in this context that fine-grained analyses of NSST responses may be of added value.

### Factors influencing adopted children's attachment representations as assessed by NSSTs

This review points to a number of promising, albeit to date not sufficiently investigated, avenues for research and/or clinical application of NSSTs with adopted children. First, although studies using a categorical approach to NSSTs have yielded inconsistent findings, studies from a more fine-grained attachment perspective suggest that adoptive parents' (mostly mothers') secure state of mind plays an important role in fostering the development of more positive IWMs in their children. However, these findings need to be interpreted with caution, as only a few studies have investigated the association between parental and child attachment representations, and none of these studies have used data-analytic procedures to warrant firm conclusions as to causality. Moreover, there is a surprising lack of studies investigating the association between paternal and child attachment representations.

Second, child and adoption characteristics such as gender and age at adoption seem to have some effect on child attachment, although specific hypotheses require further testing as findings are inconsistent across studies. For instance, whereas age at adoption yields inconsistent results, more detailed analyses, such as those of Román et al. (2012), disentangling age at adoption from variables such as time in adoptive family or pre-adoption family experience, provide promising insights into risk and protective factors for child attachment development.

Third, findings regarding the influence of child cognitive factors on attachment remain equivocal to date and thus warrant further investigation. More generally, studies using NSSTs as predictors or outcomes of adopted children's development remain relatively scarce, although such studies may yield important insights into these children's developmental trajectories.

In general, the findings of the present review should be considered preliminary, due to (a) the limited number of studies, (b) the large variety in the composition of story stem batteries as well as in coding procedures, and (c) the large variety in study designs, rendering direct comparison of results difficult. In many cases, only two or three studies included the same variables assessed at comparable time points to investigate the same research questions, so aggregate findings in the present review are few, and narrative, though not statistical, aggregation was feasible.

### Promising avenues for research and clinical application of NSSTs

Based on the findings of the present review, a number of recommendations for future research and clinical applications of NSSTs may be outlined. First, the different theoretical perspectives should be explored and investigated more thoroughly, and researchers and clinicians using NSSTs should locate themselves within these theoretical paradigms in a more explicit way.

Furthermore, empirical and clinical work in the domain of attachment should be situated as categorical, dimensional and/or fine-grained, in order to aid the reader to better grasp the diversity of knowledge available in this domain. In this regard, the findings of the present review support the added value of a fine-grained perspective on (changes in) attachment over and above a categorical or dimensional approach.

Finally, with regard to adopted children's attachment representations, much more research that is designed in theoretically, empirically, and methodologically consistent ways is needed to investigate factors influencing and influenced by child attachment. The present review's finding that NSSTs to date have been applied with considerable heterogeneity in study design and investigated variables is of particular concern and should be addressed in future research in order to enable a more systematic body of knowledge to emerge. In this regard, it is important, both as researchers and as clinicians, to remember that adverse experiences are not unique to the early lives of some adopted children. Among other children in out-of-home care, such as foster children, a subgroup has been exposed to similar early adversities, making them vulnerable to at-risk or maladaptive developmental outcomes. Without discarding the possible differences between adoption and foster care policies and practices, and fully aware of the importance of considering research findings within their specific socio-cultural context, we do also advocate a more systematic body of knowledge about the developmental trajectories of children with a history of early adversity, whether adopted or in foster care or another type of out-of-home care. The findings of the present review suggest that NSSTs, in particular, may be a fruitful avenue to pursue in research on and clinical care for such vulnerable children. Yet, an exploratory search and screening of extant research, conducted at the design stage of the present review, indicated that studies in which NSST findings were reported separately for adopted and foster children were scant, rendering it, at present, nearly impossible to disentangle possible differences between these groups.

### Limitations of the review

As discussed, the present review is based on a relatively small number of studies characterized by considerable heterogeneity in theoretical perspective as well as with regard to methodological aspects. Hence, the findings about aspects of adopted children's internal reality should be considered preliminary and be interpreted with caution.

Another limitation of the present review at design level should be mentioned. The search was conducted in a relatively small number of databases, and only English-language peer-reviewed publications were considered for inclusion, to the exclusion of possible relevant publications in other languages or gray literature.

## Conclusion

In sum, although NSST research with adopted children is still in its infancy in terms of the relative paucity of studies and the lack of consensus with regard to composition and coding of story stems, rendering direct comparison of results difficult, there is a relatively robust evidence base for NSSTs as a measure to assess and track developmental change in adopted children's attachment representations. In this regard, a fine-grained perspective on children's NSST responses has been argued to be particularly valuable in tailoring treatment to a particular child's needs and vulnerabilities and thus fostering development. Future research and clinical application of NSSTs in several avenues that have proven promising may further our knowledge and understanding of, and thus our ability to aid, these often vulnerable children. Based on the present review, we therefore strongly advocate a larger and importantly, more consistent body of cross-sectional and longitudinal studies using NSSTs, in order to investigate aspects of adopted children's internal reality in terms of attachment representations, affect and affect-regulation, and mentalization more in depth; as well as to track changes in these aspects to gain a better understanding of these children's experiences and to better tailor treatment.

## Author contributions

NV designed the study. DB and ET conducted the literature searches. NV and DB assessed study quality. All three authors contributed to data extraction and synthesis from the primary studies. NV and ET drafted the first version of the manuscript. All authors approved the final version of the manuscript.

### Conflict of interest statement

The authors declare that the research was conducted in the absence of any commercial or financial relationships that could be construed as a potential conflict of interest.

## Footnotes

^1^We opted to also include studies on early childhood as most NSSTs have been developed for children from 4 years of age.

^2^As this was not a primary focus of the present review, and owing to space limitations, we do not discuss composition of story stem batteries and NSST coding procedures in detail. Detailed information regarding these issues is available upon request.

^3^The global insecure category is a distilled sum of disorganized, insecure-ambivalent, and insecure-avoidant rates reported in primary studies, with the exception of Kocovska et al. (2012) who only reported percentages for secure and disorganized attachment classifications.

^4^This is a follow-up study of Pace and Zavattini (2011) and Pace et al. (2012), therefore only the percentages of the most recent publication are reported.

^5^Eight themes in Steele et al. (2007a).

^6^Pace et al. (2014) also coded children's responses to the MCAST to investigate attachment dimensions.

^7^As noted in the Results, there were no studies investigating the association between NSST attachment dimensions and developmental outcomes.
